# Indirect Effects of HIV Differentiated Service Delivery Programmes on Quality of Clinic Care: A Retrospective Cohort Study of Clients Starting Antiretroviral Therapy

**DOI:** 10.1097/QAI.0000000000003916

**Published:** 2026-09-11

**Authors:** Lara Lewis, Yukteshwar Sookrajh, Johan van der Molen, Thokozani Khubone, Lise Jamieson, Andrew Gray, Jennifer Anne Brown, Kwena Tlhaku, Francesca Little, Nigel Garrett, Jienchi Dorward, Reshma Kassanjee

**Affiliations:** a Centre for the AIDS Programme of Research in South Africa (CAPRISA), University of KwaZulu-Natal, Durban, South Africa;; b Department of Statistical Sciences, University of Cape Town, Cape Town, South Africa;; c eThekwini Municipality Health Unit, eThekwini Municipality, Durban, South Africa;; d Health Economics and Epidemiology Research Office (HE2RO), University of the Witwatersrand, Johannesburg, South Africa;; e South African Centre for Epidemiological Modelling and Analysis (SACEMA), Stellenbosch University, South Africa;; f Division of Pharmacology, Discipline of Pharmaceutical Sciences, University of KwaZulu-Natal, Durban, South Africa;; g Nuffield Department of Primary Care Health Sciences, University of Oxford, Oxford, United Kingdom;; h Discipline of Public Health Medicine, School of Nursing and Public Health, University of KwaZulu-Natal, Durban, South Africa;; i Desmond Tutu HIV Centre, University of Cape Town, Cape Town, South Africa; and; j Centre for Integrated Data and Epidemiological Research (CIDER), School of Public Health, University of Cape Town, Cape Town, South Africa.

**Keywords:** HIV, differentiated service delivery, antiretroviral therapy, retention in care, South Africa

## Abstract

**Background:**

Although the benefits for clients collecting antiretroviral therapy (ART) through differentiated service delivery programmes (DSD) have been described, there are limited data on whether DSD indirectly benefits clients ineligible for DSD, by allowing more clinic resources to be redirected toward them.

**Setting:**

De-identified, routinely-collected data from 112 clinics in KwaZulu-Natal, South Africa were analyzed.

**Methods:**

Using generalized linear mixed effects regression, we assessed whether the quality of care provided to clients initiating ART (and therefore ineligible for DSD) improved after increases in the proportion of clients in their clinic referred to DSD. Quality-of-care outcomes were the probability of clients having a CD4 count test, having a 6-month viral load (VL) test and being retained for 6 months.

**Results:**

Between February 2022 and September 2023, a total of 36,865 clients initiated ART. Among those in each outcome analysis, 77% had CD4 count results, 72% had 6-month VL results, and 70% were retained for 6 months. We found a positive relationship between the clinic DSD referral proportion and test completion among newly initiated clients, although this was only weakly significant for CD4 count; when DSD referrals increased from 20% to 30%, the probability of having a CD4 count and VL test increased by 0.97% (95% CI: −0.27 to 2.58%) and 1.35% (95% CI: 0.11 to 2.95%), respectively. We found no evidence of an association with DSD and retention.

**Conclusions:**

In the first study of this topic, increases in clinic DSD referrals were associated with small improvements in care. With full DSD scale-up, programmatically meaningful effects could be achieved.

## Abstract 2

Supplemental Digital Content is Available in the Text.

## INTRODUCTION

South Africa runs the largest national antiretroviral therapy (ART) program globally, with 77% of the 7.7 million people living with HIV (PLHIV) estimated to have accessed the program in 2023 alone.^1,2^ ART has been recommended for all PLHIV in accordance with national guidelines since 2016. In recent years, access to ART through the national program has been facilitated through the rollout of differentiated service delivery (DSD) models, modes of ART delivery that are designed to make ART more accessible to PLHIV than traditional clinic-based care. The 3 main DSD models endorsed by the South African National Department of Health, namely adherence clubs, facility-based pick-up points, and out-of-facility–based or external pick-up points, are supported by the Central Chronic Medicines Dispensing and Distribution (CCMDD) program.^3^ In 2023, an estimated 2.6 million PLHIV in South Africa had an active CCMDD prescription.^4^

Clients who are stable-in-care (ie, virally suppressed, without tuberculosis, not pregnant, and without an uncontrolled chronic condition) are eligible to be referred to any 1 of the 3 DSD models.^5^ Once referred, clients may collect ART from their selected pick-up point and need to only attend a clinical consultation at the clinic approximately every 6 months, whereat DSD eligibility is reassessed. Clients who are not referred to DSD are required to visit the clinic more frequently, with most visiting clinics every 2–3 months. Despite initial concerns that less frequent consultations may negatively affect the outcomes of those using DSD models, there is growing evidence that 6-monthly consultations are generally preferred by clients^6–8^ and may in fact improve clinical outcomes. In particular, improvements in retention in care and/or viral suppression among clients enrolled in DSD have been observed in randomized controlled trials^9^ and observational studies.^10–14^ Qualitative research has indicated that DSD may additionally benefit those not using DSD models, with the reduction in overall clinical consultations allowing health care workers to provide better quality of service to those attending clinics.^15–17^ However, this has not been quantitatively measured.

Measuring the effect of the DSD rollout on those not participating in the program, and not only those in it, is critical to understanding the overall effect of DSD on people on ART. In this study, we explore whether the DSD rollout has had an effect on the quality of care of PLHIV who are newly initiated on ART in the period before which they become eligible for DSD. Specifically, we aimed to measure whether increases in the proportion of clients in a clinic referred to DSD reduced overall clinic volumes, thereby improving the outcomes of newly initiated clients in the first 6 months on ART.

## METHODS

### Study Design and Setting

We performed a retrospective cohort analysis of clients initiating ART using de-identified routinely collected electronic data collected between 1 February, 2022 and 31 October, 2023 from public sector primary care clinics in the eThekwini municipality and uMgungundlovu districts of KwaZulu-Natal (KZN), South Africa. In 2022, the HIV prevalence in KZN was estimated to be 16%.^1^ The eThekwini district is densely populated, with a predominantly urban population, whereas uMgungundlovu has a mix or urban, periurban, and rural areas. DSD programs were introduced in the province from mid-2016. Since then, the DSD model allowing for ART collection through external pick-up points (such as private pharmacies and community centers) has been predominantly used. In this analysis, we have considered the effect of DSD programs using external pick-up points only because the use of other DSD models during the observation period was low (the median clinic referral to either adherence clubs or internal pick-up points was <1%). We refer to these external pick-up point models as “DSD” hereafter.

The study aims to measure the quality of care provided to clients initiating ART while they are ineligible for DSD. Newly initiated clients are not eligible for DSD until they have attained their first suppressed viral load result. In the period used for this analysis, viral loads were measured on clients on ART at 6-month and 12-months after ART initiation and annually thereafter.^18^ Thus, newly initiated clients were ineligible for DSD for the first 6 months after ART initiation.

### Data Sources and Data Management

We analyzed deidentified person-level data from TIER.Net using R 4.0 (R Foundation for Statistical Computing, Vienna, Austria) and SAS, version 9.4 (SAS Institute Inc). TIER.Net is an electronic register consisting of data on PLHIV receiving ART from South African public sector primary care clinics.^19^ It includes basic demographic data, data on visit dates to primary care clinics, referral to DSD programmes, ART start dates and regimens, prescription lengths, CD4 count and viral load measurements, and pregnancy and TB screening results.

### Outcome and Exposure Measurement

The primary outcomes measuring quality-of-care were the probability of having a CD4 count test result at ART initiation, of having a viral load test result at 6 months after ART initiation among those visiting at 6 months, and of retention for 6 months after ART initiation. CD4 count tests are required for all clients initiating ART at the time of initiation. For this outcome, we considered any CD4 count test performed 6 months before or 1 month after ART initiation as completed at ART initiation. For the 6-month viral load outcome, we included clients visiting between 5 and 9 months after ART initiation and determined whether a viral load was measured and recorded at this visit. For the retention outcome, we defined retention on ART for 6 months as not being more than 90 days late for any visit scheduled during the first 6 months on ART, whether it was owing to disengagement in care, death, or any other reason. Those who miss a visit were considered as “not retained” from the point of their last recorded visit.

Although the outcome variables were measured on a person-level, the exposure variable was measured on a clinic level. The exposure variable was the clinic-level proportion referred to DSD which varied over time. DSD referral includes all clients given a new DSD script, and not just those referred to DSD for the first time. The proportion of clients referred to DSD will effect clinic visit attendance and health care worker capacity approximately 2–4 months after referral, because clients previously on 2-month dispensing referred for DSD will not require their 2-month and 4-month clinic visits, and clients previously on 3-month dispensing will not require their 3-month visit. Thus, the DSD exposure variable was defined as the proportion of clients visiting in the period 2–4 months before the outcome measurement who were referred to DSD at any visit in the 3-month period. The value can range between 0% and 100%, although a value of 100% is unlikely as a portion of visiting clients will be ineligible.

For the analysis of CD4 count and viral load tests completion proportions, we considered only 1 DSD exposure value, that being clinic DSD proportion measured 2–4 months before the outcome measurement. However, for the analysis of retention, which was measured based on clinic attendance at several time points in the first 6 months on ART, the DSD exposure variable was time-varying. The DSD exposure value used at a scheduled visit was the DSD referral proportion measured 2–4 months before their last attended visit, as this would have informed their decision to continue on treatment until the scheduled visit.

The analysis was restricted to using data from 2022 because the DSD rollout and clinic service delivery before this was strongly affected by the COVID-19 pandemic.^20,21^ We defined the DSD exposure variable using data from April 2022 onward, to allow for the washout of 12-month DSD scripts that were temporarily allowed from May 2020 to September 2021, during the height of the COVID-19 pandemic.^22^ Outcomes were measured from August 2022 to allow for the lag between exposure and outcome measurement. In light of this and to allow sufficient follow-up time to measure the outcomes, for the CD4 count test analysis, we included clients initiated between August 2022 and September 2023, for the viral load analysis, we included clients initiated from February 2022 to January 2023 (with 6-month visits occurring in August 2022 and July 2023, respectively) and for the retention outcome we included clients initiated between August 2022 and January 2023.

In addition to measuring a DSD exposure variable, we also defined a variable quantifying clinic volumes of clients seeking ART. We hypothesized that DSD affects quality of care of newly initiated clients through their effect on clinic volumes and, as a result, staff capacity. Developing a better understanding of the association between clinic volumes and our outcomes may therefore assist in interpreting the association measured between DSD referrals and outcomes. The variable measuring clinic volumes was defined as the number of visits to a clinic in a given month over the clinic size, where clinic size was defined as the number of clients registered to receive ART in that clinic in the given month.

### Estimand

Our estimand of interest for a given outcome was the difference in probabilities (or “risk” difference) of an outcome for any 2 values of clinic DSD exposure. In this analysis, we measured the risk difference when the DSD referral proportion is set to 20% compared with 10%, and when DSD is set to 30% compared with 20%, as these are commonly observed values for DSD referrals in KZN clinics based on descriptive analysis of the data (see results section).

### Confounders

Person-level variables were not considered as confounders to the association between outcomes and exposure because characteristics of newly initiated clients, who are ineligible for DSD, are unlikely to affect DSD referrals (see Figure S1, Supplemental Digital Content, http://links.lww.com/QAI/C711). However, certain clinic-level characteristics—such as staff capacity, and skills and infrastructure—may confound the relationship: for example, better resourced clinics may have higher DSD referral proportions and provide better support to newly initiated patients. We do not have direct measures of these characteristics, and thus these variables are considered unmeasured confounders. We did however adjust for clinics as a whole in our models, discussed below.

### Statistical Analysis

We described characteristics of clients using medians, interquartile ranges (IQR), percentages, and frequencies. We plotted monthly DSD referrals and outcomes across clinics over time, and the clinic volume variable against cumulative DSD referrals in the 2–4 months before.

We used generalized linear mixed effect logistic-normal models (GLMM) to regress the odds of having a CD4 count result and the odds of having a viral load result against DSD referral proportion. Covariates included a time variable measuring months since August 2022, and clinic-specific random effects on the intercept. We included random effects to capture variation in outcomes by clinic, in the absence of measured clinic-level variables and to account for clustering of clients within clinics. Time was included to capture background trends in quality of care (for reasons other than DSD programme expansion). In addition, covariates for year quarter of ART initiation (values 1–4), participant age (in decades), and sex were included to improve precision. For the retention outcome, we used data expanded into person-month format and applied pooled GLMM to estimate the discrete-time hazard of missing a visit over a period of 1 month. Instead of including the variable measuring months since August 2022, we included a variable measuring months since ART initiation. The 6-month retention probability was derived by multiplying the complements of the estimated monthly probabilities of missing a visit over 6 months.

Next, we estimated the marginal probabilities for each outcome using numerical averaging of clinic random effects.^23^ First, we sampled 1000 random effects for each clinic from a normal distribution with mean vector zero and variance equal to the model-estimated variance of the random effect. Second, for a given DSD level, we used the realized values and the fitted fixed effect estimates to estimate 1000 conditional probabilities of the outcome and then average across the 1000 values. For the retention outcome, we obtained the 6-month probability of retention as the product of the monthly retention probabilities. Third, we obtained the probability of the outcome by taking an average of the outcome probabilities above, based on the observed distribution of covariates in the study population. The 3 steps were repeated for varying DSD referral levels. Outcome risk differences were calculated for the contrasts 20% versus 10% and 30% versus 20%. We used nonparametric bootstrapping to obtain percentile-based 95% confidence intervals (CI) for the risk differences using 500 bootstrap samples, where sampling occurred at a clinic level.

We repeated the above model building using clinic volumes as the exposure variable.

## RESULTS

**FIGURE 1.**
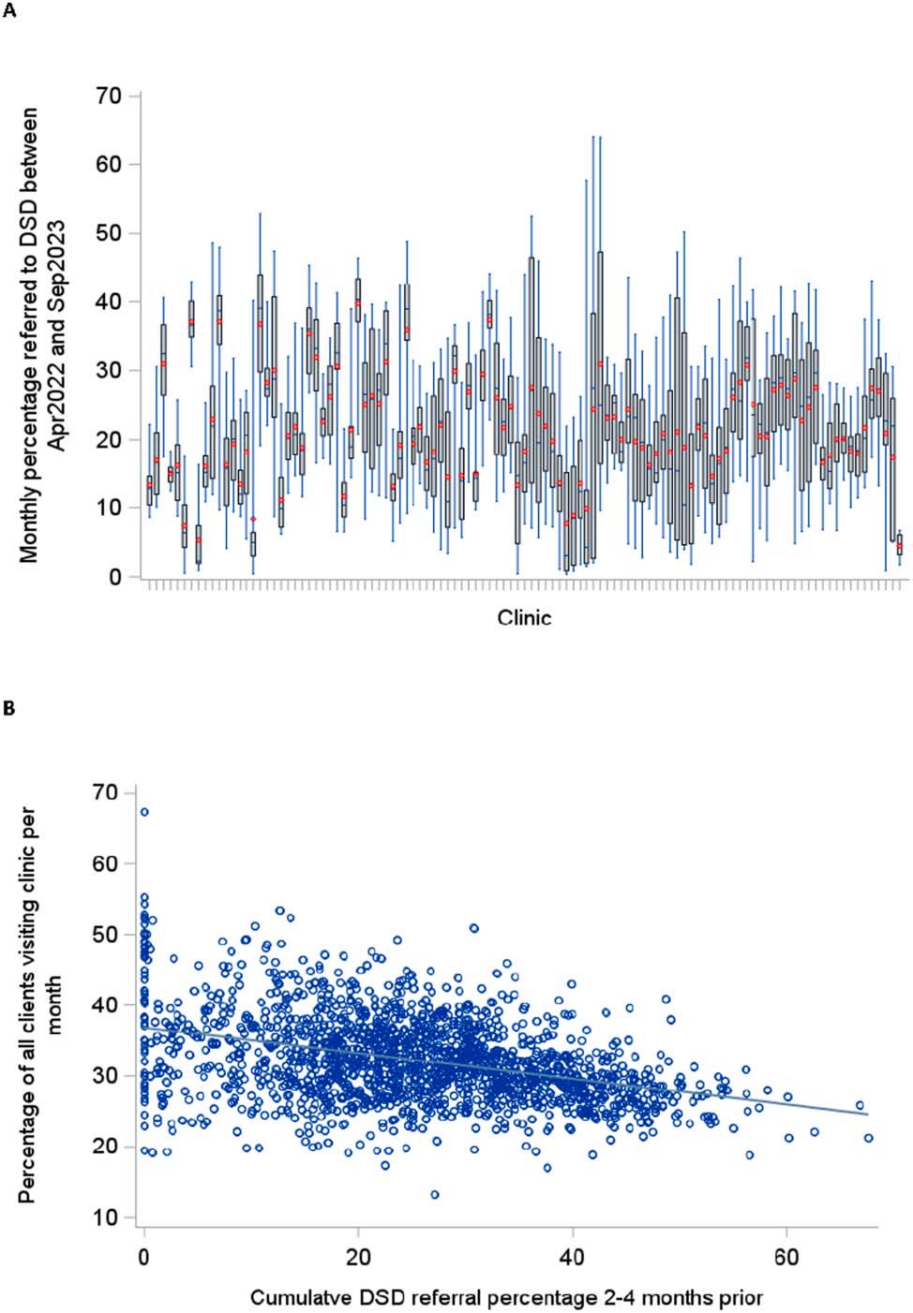
A, Box-plot of monthly DSD referral percentages per clinic with mean values displayed in red, interquartile ranges with gray boxes, and whiskers extending to minimum and maximum values; (B) percentage of all clients visiting a clinic per month against DSD referral percentage 2–4 months ago, with linear regression line in gray.

Between February 2022 and September 2023, a total of 36,865 PLHIV were initiated on ART from 112 clinics. The median (IQR) age of these clients was 32 (26–39) years, 63% were female, and most (97%) were initiated on a dolutegravir-based regimen (Table 1). DSD referrals varied across the clinics (Fig. 1A); the median (IQR) monthly clinic DSD referral proportion across the observation period was 23% (15%–32%). There was also variability in DSD referrals within clinics; the difference in the highest and lowest monthly DSD referral of a clinic during the observation exceeded 15% in 73% of clinics. The scatterplot of monthly clinic volumes against the cumulative percentage referred to DSD 2–4 months ago, showed a negative trend between DSD referral and clinic volumes, with much variation in the level of clinic volumes per DSD referral level (Fig. 1B).

**TABLE 1.** Sample Description

	Median (IQR) or N (%)
Characteristics of clients initiating ART between Feb 2022–Sep 2023 (N = 36,865)	
Age in years	32 (26–39)
Female	23,243 (63)
Attends clinics in eThekwini district	24,972 (68)
Initiated on antiretroviral therapy containing dolutegravir	35,681(97)
Characteristics of clinics attended by newly initiated clients (N = 112)*	
Size (ie, number of clients active on ART in clinic)	2822 (1820–4584)
Located in eThekwini district	58 (52%)
Proportion of clinic clients referred to external pick-up points	16% (11%–22%)
Proportion of clinic clients referred to internal pick-up points	1% (0%–5%)
Proportion of clinic clients referred to adherence clubs	0% (0%–3%)

* As of Apr 2022, month from which DSD referrals are analyzed.

**FIGURE 2.**
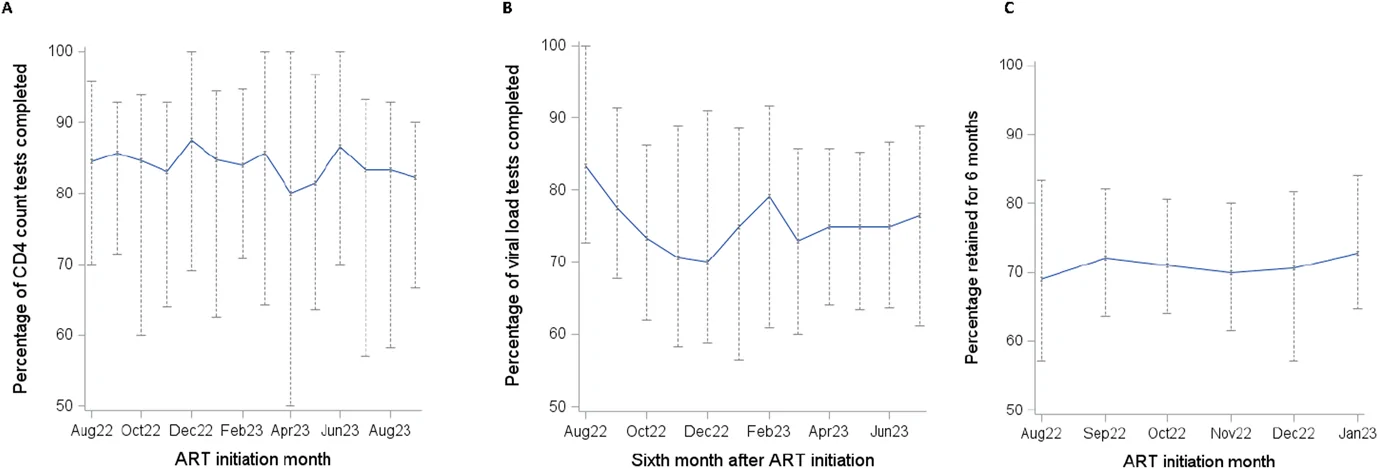
Median ( line) and interquartile range (gray bars) of monthly clinic percentages of (A) CD4 count test completion, (B) 6-month viral load test completion (among those attending their 6-month visit), and (C) 6-month retention among clients initiated on ART.

The percentage of clients initiated on ART between August 2022 and September 2023 with a CD4 count test completed was 77% (19,031/24,778). Of those, most (94%) had the test performed on the same day as initiation. The CD4 count completion percentages varied by clinic (Fig. 2A); the median (IQR) clinic CD4 count test completion percentage over the period was 81% (68%–90%). Results from the GLMM suggested a positive relationship between DSD referrals and CD4 count test completion probability, although this association was small, and only weakly significant; when recent clinic DSD referrals increased from 10% to 20% and from 20% to 30%, the probability of having a CD4 count test result increased by 0.99% (95% CI: −0.27 to 2.72%) and 0.97% (95% CI: −0.27 to 2.58%), respectively (Table 2, see Supplemental Digital Content, Table 1, http://links.lww.com/QAI/C711). In line with these findings, we found a significantly negative relationship between clinic volumes and CD4 count test completion, with test completion probability decreasing by approximately 2% for a 10% increase in the percentage of the clinic attending a monthly clinic visit (Table 2, Supplemental Digital Content, Table 2, http://links.lww.com/QAI/C711).

**TABLE 2.** Estimated Marginal Effect of Increases in Clinic DSD Referrals and Clinic Volumes on CD4 Count Test Completion, 6-Month Viral Load Test Completion, and 6-Month Retention Among Clients Who Were Newly Initiated on ART

Sample Description	Outcome Variable	#clients (#clinics)	Exposure Variable	Risk Difference (95% CI) with Change from 10% to 20%	Risk Difference (95% CI) with Change from 20% to 30%
Initiations from Aug 2022 to Sep 2023	CD4 count test completed	24,778 (112)	DSD referral	0.99% (−0.27% to 2.72%)	0.97% (−0.27% to 2.58%)
			Clinic volumes	−2.14% (−3.71% to −0.35%)	−2.27% (−4.21% to −0.35%)
Initiations from Feb 2022 to Jan 2023	6-month viral load test completed	16,795 (112)	DSD referral	1.38% (0.11% to 3.11%)	1.35% (0.11% to 2.95%)
			Clinic volumes	−0.94% (−2.28% to 0.67%)	−0.97% (−2.43% to 0.66%)
Initiations from Aug 2022 to Jan 2023	Retained for 6 mo	11,542 (112)	DSD referral	0.17% (−0.45% to 1.53%)	0.17% (−0.45% to 1.48%)
			Clinic volumes	1.60% (−0.19% to 4.27%)	1.55% (−0.19% to 3.95%)

Among clients initiated on ART between February 2022 to January 2023 and who attended a visit approximately 6 months after their ART initiation, 72% (12,059/16,795) had a viral load test result at this visit. The median (IQR) time to viral load test completion from ART initiation was 188 (171–201) days. The proportion of clients with a viral load test completed varied by clinic (Fig. 2B); the median (IQR) clinic viral load test completion percentage over the period was 73% (66%–83%). We found evidence of a small positive relationship between DSD referral and the probability of viral load test completion; when DSD referral before the visit increased from 10% to 20% and from 20% to 30%, the probability of having a viral load test result increased by 1.38% (95% CI: 0.11 to 3.11%) and 1.35% (95% CI: 0.11 to 2.95%), respectively (Table 2, Supplemental Digital Content, Table 1, http://links.lww.com/QAI/C711). The association between clinic volumes and the probability of having a viral load test result was negative, but not statistically significant.

Among the 11,542 clients initiated on ART between August 2022 and January 2023, 3450 (30%) were more than 90 days late for at least 1 of their scheduled visits in the first 6 months. Approximately, 41% of these events occurred within 1 month of ART initiation. The proportion retained for the first 6 months varied by clinic (Fig. 2C). Results from the GLMM showed no evidence of an association between DSD referrals and/or clinic volumes and the 6-month probability of missing a visit (Table 2, Supplemental Digital Content, http://links.lww.com/QAI/C711, Table 1 and 2). The estimated change in 6-month retention for an increase in DSD referrals from 10% to 20% and 20%–30% was 0.17% (−0.45%; 1.53%) and 0.17% (−0.45%; 1.48%), respectively.

## DISCUSSION

In this study, we analyzed longitudinal data from 112 clinics, examining whether variability in each clinic's DSD referrals over time was associated with changes in CD4 count and viral load test completion and 6-month retention of newly initiated clients. We hypothesized that increases in referrals to DSD would lower the number of clients visiting clinics, thereby allowing more resources to be allocated to new patients, improving their level of care and outcomes. Although our results showed a negative correlation between clinic volumes and DSD referrals, it was not perfectly linear, highlighting that there are several determinants of clinic volumes over and above DSD referrals. We found that 10% increases in clinic DSD referrals were associated with small improvements in test completion probabilities, but there was no evidence of an association with retention.

There has been little research conducted on the potential effect of DSD for ART on clients who are ineligible for DSD, against which our findings can be benchmarked. A recent study in Mozambique found that implementation of 8 different types of DSD models in the country improved population-level 12-month retention after ART initiation by 24.5%.^24^ However, the use of a 12-month outcome period in this analysis meant that the sample included a mix of clients who were enrolled in DSD, not enrolled but eligible, or ineligible, contrasting with our sample that included only those who were ineligible. In addition, although not reported, the magnitude of the DSD program expansion in Mozambique was likely large given that 8 models were used. This analysis is restricted to data from 2022 to 2023 during which increases in clinic DSD referrals (although greater than 15% in most clinics) was unlikely to have been as large, and it is possible that the effects of reduced clinic visit frequency on retention will only be observed with greater scale up. Recent changes to the South African ART program, namely the widening of DSD eligibility requirements to include clients from 4 months after ART initiation^25^ and the expansion of 6-month multimonth dispensing,^26^ are two avenues through which this can be achieved.

We chose retention as an outcome, hypothesizing that the higher DSD referrals would improve client clinic experience and/or allow those in need of support more time with health care workers. However, particularly for clients newly initiating ART, client-specific factors, such as difficulty accepting the HIV diagnosis, ART pill burden, and side effects, may outweigh or negate the benefits of improvements in clinic experiences.^27^ Alternative outcome variables, including client waiting times, consultation times, and the number of clients initiated on ART should also be considered for future studies. Qualitative research to understand how additional time is prioritized within primary care clinics would be needed to complement this quantitative work.

Key strengths of our analysis include the large number of clients included in the analysis, and the longitudinal nature of our data. Nevertheless, our findings must be interpreted considering the study limitations. First, confounding and/or modification by clinic characteristics are a key concern in the analysis. We used a random effect on the intercept in regression analyses, thereby allowing for expected values of the outcomes at a given DSD level to vary by clinic. Although this measure will fail to measure short-term changes in clinic qualities, the analysis was conducted over a relatively short observation period, during which large changes were unlikely. Second, the effect of possible inaccuracies in TIER.Net data needs to be considered when interpreting these findings. Although in recent years the quality of TIER.Net data has improved, laboratory measurements captured in TIER.Net are known to be incomplete.^28,29^ Therefore, we could not determine whether a missing laboratory test result in TIER.Net was because of missed test completion or incomplete data capture, although both are measures of service quality. Finally, the definition of retention used in our study, requiring 100% attendance at all visits in the first 6 months on ART, was stringent and future work should explore a wider range of retention definitions.

## CONCLUSIONS

This study was the first known analysis of the effect of DSD on clients initiating ART who are ineligible for DSD. Our findings suggest that modest changes in DSD referrals have a small effect on the quality of care received by these clients, but that full DSD scale-up could lead to meaningful improvements in care. Measuring the effect of DSD on other groups of ineligible clients, while it presents methodological challenges, will be important to understand the overall effect of DSD. A wider range of outcomes and larger increases in DSD referrals than observed in this study should be considered before the effect of DSD programs on ineligible clients can be fully understood.

## Supplementary Material
